# Residential segregation and late-stage colorectal cancer in the United States: a population-based study of 1.2 million adults

**DOI:** 10.1093/aje/kwaf285

**Published:** 2025-12-24

**Authors:** Eduardo J Santiago-Rodríguez, Justin S White, Zinzi D Bailey, Isabel E Allen, Robert A Hiatt, Salma Shariff-Marco

**Affiliations:** Department of Epidemiology and Biostatistics, University of California, San Francisco, San Francisco, CA, United States; Department of Health Law, Policy and Management, Boston University School of Public Health, Boston, MA, United States; Division of Epidemiology and Community Health, University of Minnesota School of Public Health, Minneapolis, MN, United States; Department of Epidemiology and Biostatistics, University of California, San Francisco, San Francisco, CA, United States; Department of Epidemiology and Biostatistics, University of California, San Francisco, San Francisco, CA, United States; Helen Diller Family Comprehensive Cancer Center, University of California, San Francisco, San Francisco, CA, United States; Department of Epidemiology and Biostatistics, University of California, San Francisco, San Francisco, CA, United States; Helen Diller Family Comprehensive Cancer Center, University of California, San Francisco, San Francisco, CA, United States; Greater Bay Area Cancer Registry, San Francisco, CA, United States

**Keywords:** institutionalized racism, residential segregation, colorectal cancer, stage at diagnosis

## Abstract

We examined the association between residential segregation and late-stage colorectal cancer (CRC) in the United States. The restricted-use United States Cancer Statistics database was used to identify all CRC cases diagnosed during 2009 to 2017. Late-stage CRC was determined according to the presence of distant involvement of the tumor at diagnosis. Residential segregation was measured at the county level by the Index of Concentration at the Extremes based on income, race/ethnicity, and its combination, using the 2013-2017 American Community Survey data. Multilevel logistic regression models accounting for clustering at counties were fit. Analyses were stratified by race/ethnicity, sex, and age. Overall, patients residing in counties with a high concentration of least advantaged residents had increased odds of late-stage CRC compared to their counterparts residing in counties with a high concentration of most advantaged people. These findings were observed on all measures of residential segregation, with clear gradients for economic and racialized economic segregation. In stratified analyses, stronger associations were observed among racial/ethnic minoritized people and younger age groups; results did not differ by sex. These findings underscore the role of institutionalized racism as a contributor to health inequities, such that laws and policies driving residential segregation may impact timely preventive care.

## Introduction

During the last decades, improvements in screening uptake, risk reduction behaviors, and advances in treatment have resulted in lowering colorectal cancer (CRC) incidence and mortality rates in the United States.^1^ Nevertheless, a total of 154 270 new cases and 52 900 deaths are expected to occur in 2025.^2^ Research suggests that approximately 60% of CRC-related deaths could be avoided with screening,^3^ which translates to over 30 000 preventable deaths based on the 2025 projection. These numbers situate CRC as the third most common cancer in women and men, and as the second leading overall cause of cancer mortality.^2^ These numbers also represent deficiencies in the way preventive care services are delivered in the United States, and how healthcare systems and public health initiatives have failed to ensure equitable benefit of established cancer control strategies across populations, especially to groups facing the largest burden of the disease (ie, racial/ethnic minoritized groups, uninsured people, foreign-born individuals, and those living in rural areas).^4^^-^^6^

Access to health care is necessary to obtain the services that allow people to avoid or manage a CRC diagnosis.^7^ Although access to health care is often perceived as being determined by individual characteristics and dependent on socioeconomic status (eg, individuals’ education, employment status, income), calls have been made over the years emphasizing the role of upstream factors to health care in the context of achieving health equity across the cancer continuum.^8^^-^^11^ Specifically, there is a need for assessing and addressing elements of the social and built environments in areas where individuals reside.^9^ One key feature is residential segregation, rooted in racism, embodying the disenfranchisement of communities by institutions and policies in the United States.^12^^,^^13^ These communities, predominantly populated by racial/ethnic minoritized groups, are characterized by public and private disinvestment, making it difficult for individuals to engage with health care systems and receive high-quality preventive care.^14^^-^^16^ Residential segregation is a manifestation of institutional racism, which refers to adverse discriminatory policies and practices carried out by institutions based on racialized group membership.^14^^,^^17^

In this study, we examined the association between residential segregation and late-stage diagnosis of CRC in the United States. Previous research evaluating this association has produced mixed findings, with some reporting lower odds of advanced CRC in more segregated areas and others finding no association or higher risk.^18^^-^^22^ Also, prior work focused mostly on racial residential segregation,^18^^-^^21^ included solely non-Hispanic Black (NHB) and non-Hispanic White (NHW) individuals,^20^^,^^22^ used samples of patients,^18^^-^^22^ or employed ecological analyses.^21^ This study, on the other hand, used a measure of residential segregation that included information on race/ethnicity, income, and its combination, generating metrics of racial, economic, and racialized economic residential segregation and providing a more comprehensive assessment of the social environment. Also, by using a national data source with complete coverage (ie, 100% of adults with CRC), we leverage a rich dataset and the opportunity for evaluating different versions of racial and racialized economic segregation, consisting of comparisons of not only NHW and NHB racial groups but also Hispanic/Latino and Asian American/Pacific Islander (AAPI) groups, and all non-NHW group. Additionally, we examined differences by race/ethnicity, sex, and age.

## Methods

### Data source and study population

We obtained information from the restricted-use United States Cancer Statistics (USCS) database, accessed through a federal statistical data center. In the United States, it is mandated by law that all new cancer diagnoses be reported to cancer registries in states and territories. The USCS database combines data from the CDC's National Program of Cancer Registries (NPCR) and the NCI's SEER Program. NPCR supports cancer registries in 46 states, the District of Columbia, and US territories, while SEER supports 21 cancer registries with detailed data in selected geographic areas and covers about 48% of the US population.^23^^,^^24^ Together, they provide comprehensive surveillance of cancer incidence covering the entire US population.^25^ The study population were all individuals (≥20 years) with a primary CRC diagnosis during the period of 2009 to 2017. We used the 2019 submission that includes data for 50 states and the District of Columbia.^26^ County of residence at diagnosis was used to link measures of residential segregation calculated using the methods explained below.

### Exposure

Residential segregation was measured at the county level by the Index of Concentration at the Extremes (ICE) using the 2013-2017 American Community Survey (ACS) data, following procedures described by Krieger and colleagues.^27^^,^^28^ Created initially as a measure of economic polarization, ICE has been modified in recent years to include racial/ethnic data and produce metrics of economic, racial, and racialized economic residential segregation.^27^^-^^29^ In this study, ICE was calculated to measure the extent to which individuals’ residence at diagnosis was located in counties of concentrated advantage or disadvantage.

ICE measures were calculated using the formula: ICE_i_ = (*A*_i_-*P*_i_)/*T*_i_, where for a given county i, *A*_i_ represents the number of households in the most advantaged group, *P*_i_ represents the number of households in the least advantaged group, and *T*_i_ represents the total count of households with known income and race/ethnicity information. For economic segregation, households with incomes in the top quintile of the US distribution were considered the advantaged group and households with incomes in the bottom quintile of the US distribution were considered the disadvantaged group. For racial segregation, NHW individuals comprised the advantaged group, and the disadvantaged group was defined in multiple ways corresponding to the different racial/ethnic minoritized groups: (1) people of color (POC) or all non-NHW, (2) NHB, (3) Hispanic/Latino, and (4) AAPI. For racialized economic segregation, four versions were evaluated as well, and households with White individuals reporting income in the top quintile of the US distribution were considered the most advantaged group, and households with racial/ethnic minoritized individuals reporting income in the bottom quintile of the US distribution were considered the most disadvantaged group [again, (1) POC or all non-NHW, (2) NHB, (3) Hispanic/Latino, and (4) AAPI].

ICE measures range from −1, representing counties with 100% of the population in the most disadvantaged group, to 1, representing counties with 100% of the population in the most advantaged group. For this study, ICE measures were further subdivided into quintiles (Q1—least advantaged, Q5—most advantaged) and weighted using the total population in the United States in 2013-2017 ACS data.^22^

### Outcome

Late-stage CRC was determined by the presence of distant involvement (node or site) of the tumor at diagnosis. We used the Merged Summary Stage variable in USCS, in which cases diagnosed in 2004 or later are classified using the Derived SEER Summary Stage 2000 variable. These staging criteria characterize cancers as localized, regional, distant, or unknown stage. Localized cancer is confined to the primary site; regional cancer has spread directly beyond the primary site (regional extension) or to regional lymph nodes; and distant cancer has spread to other organs (distant extension) or remote lymph nodes.^30^ Only cases with distant stage were considered a late-stage diagnosis.

### Covariates

Other variables included in the analyses were: age (continuous and 20-49, 50-75, > 75 years), sex (male, female), race/ethnicity (NHW, NHB, Hispanic/Latino, AAPI, American Indian/Alaska Native (AIAN), and unknown in USCS, hereafter referred to as White, Black, Latino, AAPI, AIAN, and unknown), census region (Midwest, Northeast, South, West), and year of diagnosis (2009-2017).

### Statistical analysis

We used descriptive statistics to characterize the study population. Multilevel logistic regression models were fit to evaluate the relationship between residential segregation and late-stage CRC, accounting for clustering of individuals within counties. We estimated unadjusted regressions and adjusted regressions that included age at diagnosis, sex, race/ethnicity, census region, and year of diagnosis as fixed effects. All models included county random effects. Analyses were conducted for all individuals and were also stratified by race/ethnicity. Additional stratified analyses were performed for sex and age as potential effect modifiers. Results are presented as odds ratios (OR) and 95% CIs.

In the initial descriptive analysis, 97 687 cases (7%) had unknown stage. Those cases were excluded from the main analysis but included in sensitivity analyses. Frist, we conducted a descriptive analysis to compare the characteristics of patients by stage at diagnosis, defined as not late, late, and unknown. Second, we repeated the main analysis and evaluated two different scenarios: (1) all unknown stage cases classified as not late and (2) all unknown stage cases classified as late.

In this study, we implemented multilevel logistic regression to obtain OR as measure of association and compare our findings with prior studies. Given that ORs can overestimate effect sizes when the outcome is common,^31^ we conducted an additional sensitivity analysis by running adjusted models using Poisson regression with robust variance estimation. Results are presented as prevalence ratios (PRs) with 95% CI and are included in the supplementary material.

Statistical significance was set at $\alpha$ < 0.05. Analyses were conducted using Stata (Version 16.1; Stata Corp, College Station, TX), and figures were created using R (Version 4.2.1; R Foundation for Statistical Computing, Vienna, Austria).

The Institutional Review Board at the University of California, San Francisco approved this study as exempt (protocol 21-35 124).

## Results

A total of 1 241 892 cases were included in the analysis; mean age at diagnosis was 67 years (SD, 14 years), 53% were men, 75% were White, and 21% of cases had a late-stage diagnosis (Table 1). The distribution of measures of residential segregation is presented graphically in Figures S1-S3.

**Table 1:** Characteristics of individuals with colorectal cancer by race/ethnicity in the United States, 2009-2017.

**Characteristics**	**All, *n* = 1 241 892**	**White, *n* = 928 382**	**Black, *n* = 150 657**	**Latino, *n* = 102 016**	**Asian American/Pacific Islander, *n* = 45 503**	**American Indian/Alaska Native, *n* = 8092**
Age at diagnosis; mean (SD)	66.6 (13.9)	67.8 (13.8)	63.5 (13.1)	62.6 (14.3)	64.0 (13.9)	62.2 (13.2)
Age at diagnosis; *n* (%)						
20-49 y	134 312 (10.8)	87 920 (9.5)	19 618 (13)	17 738 (17.4)	6574 (14.4)	1285 (15.9)
50-75 y	747 403 (60.2)	542 830 (58.5)	101 816 (67.6)	63 480 (62.2)	28 859 (63.4)	5472 (67.6)
> 75 y	360 177 (29)	297 632 (32.1)	29 223 (19.4)	20 798 (20.4)	10 070 (22.1)	1335 (16.5)
Sex; *n* (%)						
Male	652 823 (52.6)	489 516 (52.7)	75 692 (50.2)	55 337 (54.2)	24 044 (52.8)	4216 (52.1)
Female	589 069 (47.4)	438 866 (47.3)	74 965 (49.8)	46 679 (45.8)	21 459 (47.2)	3876 (47.9)
Race/ethnicity; *n* (%)						
White	928 382 (74.8)					
Black	150 657 (12.1)					
Latino	102 016 (8.2)					
Asian American/Pacific Islander	45 503 (3.7)					
American Indian/Alaska Native	8092 (0.7)					
Unknown	7242 (0.6)					
Census region; *n* (%)						
Midwest	286 527 (23.1)	244 595 (26.4)	27 735 (18.4)	7039 (6.9)	3876 (8.5)	1461 (18.1)
Northeast	244 539 (19.7)	192 609 (20.8)	24 951 (16.6)	17 138 (16.8)	8304 (18.3)	303 (3.7)
South	462 892 (37.3)	326 572 (35.2)	85 363 (56.7)	38 922 (38.2)	7151 (15.7)	2600 (32.1)
West	247 934 (20)	164 606 (17.7)	12 608 (8.4)	38 917 (38.2)	26 172 (57.5)	3728 (46.1)
Year of diagnosis; *n* (%)						
2009	140 454 (11.3)	107 631 (11.6)	16 766 (11.1)	9994 (9.8)	4503 (9.9)	790 (9.8)
2010	136 766 (11)	103 960 (11.2)	16 610 (11)	10 035 (9.8)	4635 (10.2)	823 (10.2)
2011	136 360 (11)	103 108 (11.1)	16 462 (10.9)	10 400 (10.2)	4784 (10.5)	826 (10.2)
2012	135 422 (10.9)	102 076 (11)	16 354 (10.9)	10 571 (10.4)	4776 (10.5)	822 (10.2)
2013	136 734 (11)	102 268 (11)	16 748 (11.1)	11 062 (10.8)	5014 (11)	868 (10.7)
2014	138 467 (11.2)	102 769 (11.1)	16 991 (11.3)	11 713 (11.5)	5267 (11.6)	891 (11)
2015	139 231 (11.2)	102 703 (11.1)	17 080 (11.3)	12 356 (12.1)	5274 (11.6)	993 (12.3)
2016	139 580 (11.2)	102 394 (11)	16 976 (11.3)	12 710 (12.5)	5613 (12.3)	1048 (13)
2017	138 878 (11.2)	101 473 (10.9)	16 670 (11.1)	13 175 (12.9)	5637 (12.4)	1031 (12.7)
ICE–Income; *n* (%)						
Q1	278 787 (23.1)	210 676 (23.4)	46 079 (30.9)	16 064 (15.9)	1921 (4.3)	2990 (39)
Q2	257 457 (21.3)	197 881 (22)	31 005 (20.8)	21 302 (21)	4075 (9.1)	1824 (23.8)
Q3	234 472 (19.4)	172 578 (19.2)	30 188 (20.2)	22 619 (22.3)	6548 (14.6)	1152 (15)
Q4	220 910 (18.3)	157 694 (17.5)	21 829 (14.6)	25 413 (25.1)	13 519 (30.1)	871 (11.4)
Q5	217 447 (18)	160 032 (17.8)	20 264 (13.6)	15 913 (15.7)	18 879 (42)	822 (10.7)
ICE–Race, White–People of Color; *n* (%)						
Q1	204 950 (17)	85 518 (9.5)	44 633 (29.9)	51 087 (50.4)	20 684 (46)	1714 (22.4)
Q2	217 338 (18)	131 829 (14.7)	44 393 (29.7)	25 624 (25.3)	12 808 (28.5)	1052 (13.7)
Q3	247 038 (20.4)	188 044 (20.9)	35 912 (24)	13 594 (13.4)	5956 (13.3)	2154 (28.1)
Q4	251 168 (20.8)	217 462 (24.2)	184,37 (12.3)	8045 (7.9)	4089 (9.1)	1825 (23.8)
Q5	288 579 (23.9)	276 008 (30.7)	5990 (4)	2961 (2.9)	1405 (3.1)	914 (11.9)

**Table 1:** Continued

**Characteristics**	**All, *n* = 1 241 892**	**White, *n* = 928 382**	**Black, *n* = 150 657**	**Latino, *n* = 102 016**	**Asian American/Pacific Islander, *n* = 45 503**	**American Indian/Alaska Native, *n* = 8092**
ICE–Race, White–Black; *n* (%)						
Q1	225 476 (18.7)	98 452 (11)	68 626 (46)	39 622 (39.1)	16 211 (36.1)	1249 (16.3)
Q2	225 205 (18.6)	140 167 (15.6)	36 000 (24.1)	30 430 (30)	15 844 (35.3)	1141 (14.9)
Q3	222 142 (18.4)	168 575 (18.8)	25 153 (16.8)	18 353 (18.1)	6770 (15.1)	1827 (23.9)
Q4	254 344 (21)	221 674 (24.7)	15 097 (10.1)	9405 (9.3)	4614 (10.3)	2256 (29.5)
Q5	281 906 (23.3)	269 993 (30)	4489 (3)	3501 (3.5)	1503 (3.3)	1186 (15.5)
ICE- Race, White–Latino; *n* (%)						
Q1	203 064 (16.8)	94 223 (10.5)	26 225 (17.6)	58 351 (57.6)	21 411 (47.6)	1360 (17.8)
Q2	222 340 (18.4)	133 264 (14.8)	50 789 (34)	22 534 (22.2)	12 697 (28.3)	1598 (20.9)
Q3	244 608 (20.2)	182 920 (20.4)	41 291 (27.6)	11 496 (11.4)	5697 (12.7)	1806 (23.6)
Q4	255 131 (21.1)	217 401 (24.2)	24 142 (16.2)	6573 (6.5)	3783 (8.4)	1942 (25.4)
Q5	283 930 (23.5)	271 053 (30.2)	6918 (4.6)	2357 (2.3)	1354 (3)	953 (12.4)
ICE–Race, White–Asian American/Pacific Islander; *n* (%)						
Q1	205 433 (17)	88 599 (9.9)	37 193 (24.9)	50 849 (50.2)	25 943 (57.7)	1536 (20.1)
Q2	228 865 (18.9)	139 215 (15.5)	49 969 (33.5)	27 029 (26.7)	9357 (20.8)	1539 (20.1)
Q3	231 276 (19.1)	175 676 (19.5)	35 526 (23.8)	12 033 (11.9)	4995 (11.1)	1728 (22.6)
Q4	253 966 (21)	218 756 (24.3)	20 429 (13.7)	8297 (8.2)	3370 (7.5)	1815 (23.7)
Q5	289 533 (24)	276 615 (30.8)	6248 (4.2)	3103 (3.1)	1277 (2.8)	1041 (13.6)
ICE–Race, White–People of Color + Income; *n* (%)						
Q1	229 157 (19)	108 283 (12.1)	60 569 (40.6)	44 848 (44.3)	11 891 (26.5)	2409 (31.5)
Q2	248 136 (20.5)	172 972 (19.2)	40 122 (26.9)	21 300 (21)	10 422 (23.2)	1771 (23.1)
Q3	262 295 (21.7)	223 486 (24.9)	20 888 (14)	10 775 (10.6)	4212 (9.4)	1492 (19.5)
Q4	245 465 (20.3)	202 863 (22.6)	15 582 (10.4)	14 010 (13.8)	10 569 (23.5)	1102 (14.4)
Q5	224 020 (18.5)	191 257 (21.3)	12 204 (8.2)	10 378 (10.2)	7848 (17.5)	885 (11.6)
ICE–Race, White–Black + Income; *n* (%)						
Q1	245 740 (20.3)	138 789 (15.5)	72 399 (48.5)	26 457 (26.1)	5430 (12.1)	1615 (21.1)
Q2	251 182 (20.8)	169 411 (18.9)	30 714 (20.6)	32 970 (32.6)	14 527 (32.3)	1926 (25.2)
Q3	258 508 (21.4)	215 454 (24)	19 518 (13.1)	14 044 (13.9)	6198 (13.8)	1860 (24.3)
Q4	225 124 (18.6)	189 394 (21.1)	13 471 (9)	13 205 (13)	6509 (14.5)	1293 (16.9)
Q5	227 903 (18.9)	185 277 (20.6)	13 214 (8.9)	14 615 (14.4)	12 270 (27.3)	965 (12.6)
ICE–Race, White–Latino + Income; *n* (%)						
Q1	215 686 (17.8)	111 294 (12.4)	36 643 (24.5)	52 289 (51.6)	12 096 (26.9)	2141 (28)
Q2	277 309 (22.9)	207 562 (23.1)	44 725 (29.9)	12 642 (12.5)	8817 (19.6)	2224 (29)
Q3	258 571 (21.4)	205 331 (22.8)	30 479 (20.4)	14 425 (14.2)	5430 (12.1)	1436 (18.8)
Q4	233 905 (19.4)	190 060 (21.1)	21 014 (14.1)	11 654 (11.5)	8820 (19.6)	968 (12.6)
Q5	223 602 (18.5)	184 614 (20.5)	16 504 (11.1)	10 301 (10.2)	9779 (21.8)	890 (11.6)
ICE–Race, White–Asian American/Pacific Islander + Income; *n* (%)						
Q1	245 643 (20.3)	143 210 (15.9)	42 983 (28.8)	40 763 (40.2)	14 667 (32.6)	2814 (36.7)
Q2	253 379 (21)	186 311 (20.7)	38 437 (25.7)	20 087 (19.8)	5647 (12.6)	1557 (20.3)
Q3	251 453 (20.8)	199 049 (22.1)	30 862 (20.7)	13 089 (12.9)	5559 (12.4)	1442 (18.8)
Q4	235 252 (19.5)	187 798 (20.9)	20 533 (13.8)	14 466 (14.3)	10 054 (22.4)	1021 (13.3)
Q5	223 346 (18.5)	182 493 (20.3)	16 550 (11.1)	12 906 (12.7)	9015 (20.1)	825 (10.8)
Stage at diagnosis; *n* (%)						
Late	263 782 (21.2)	192 058 (20.7)	37 493 (24.9)	22 581 (22.1)	9064 (19.9)	1893 (23.4)
Not late	978 110 (78.8)	736 324 (79.3)	113 164 (75.1)	79 435 (77.9)	36 439 (80.1)	6199 (76.6)

For economic segregation overall, 64% of the study population resided in less advantaged counties (Q1-Q3; Figure S1). For all versions of racial segregation, a lower percentage of people (range, 55%-56%) resided in less advantaged counties (Figure S2). Across versions of racialized economic segregation, a more balanced distribution was observed, with the percentage in less advantaged counties closer to 60% (range, 61%-62%; Figure S3).

Measures of residential segregation varied by race/ethnicity. For economic segregation, White individuals had a similar distribution as the overall population, with 65% residing in less advantaged quintiles (Q1-Q3). This percentage was much higher among Black (72%) and AIAN (78%) individuals, but lower among Latino (59%) and AAPI (28%) individuals (Figure S1). There was a consistent distribution of each racial/ethnic group's percentage residing in less disadvantaged counties across the different versions of racial segregation. Less than half of White individuals resided in less advantaged areas (range, 45%-46%), whereas a majority of racial/ethnic minoritized groups did: Black (79%-87%), Latino (87%-91%), AAPI (87%-90%), and AIAN (55%-64%; Figure S2). For racialized economic segregation, compared to racial segregation, higher percentages of White (56%-59%) and AIAN (71%-76%) individuals resided in less advantaged areas, compared with Black (75%-82%), Latino (73%-78%), and AAPI (58%-59%) individuals (Figure S3).

When evaluating the association between residential segregation and late-stage CRC in the different models, we observed substantial similarity across the four versions of both racial segregation and racialized economic segregation. Therefore, we only present findings of White-POC measures here. Results for the other versions (White-Black, White-Latino, and White-AAPI) are presented in Figures S4-S9.

### Economic segregation

**Figure 1:**
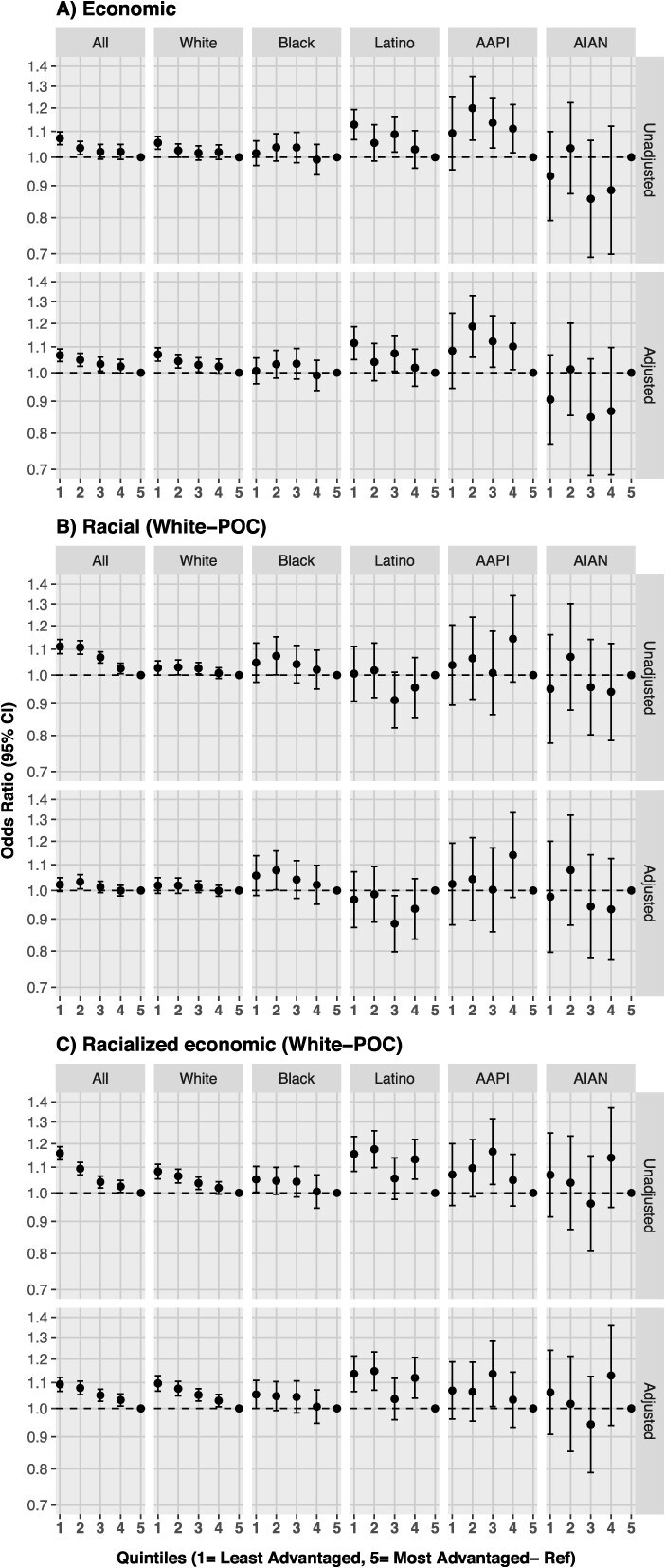
Odds of late-stage colorectal cancer by quintiles of residential segregation, overall and stratified by race/ethnicity in the United States, 2009-2017. Note: Residential segregation is based on the ICE. Panel A uses a measure of economic segregation, panel B uses a measure of racial segregation (White–people of color), and panel C uses a measure of racialized economic segregation (White–people of color). Quintile 1—least advantaged, quintile 5—most advantaged (reference). Adjusted models included: Age at diagnosis, sex, race/ethnicity, census region, and year of diagnosis. Abbreviation: ICE, Index of Concentration at the Extremes.

For economic segregation, results of unadjusted and adjusted models were very similar. In the adjusted analysis for all individuals, a gradient was observed such that those residing in less advantaged counties had increased odds of a late-stage diagnosis compared to persons residing in the most advantaged counties (OR [95% CI]: Q1, 1.07 [1.04-1.09]; Q2, 1.05 [1.02-1.07]; Q3, 1.03 [1.01-1.06]; Q4, 1.02 [1.00-1.05]; Q5, reference; Figure 1A).

In the economic segregation analysis stratified by race/ethnicity, results for White individuals resembled the results for all individuals, as they comprised the majority of the study population. Among Black individuals, the gradient was less clear, and all estimates included the null. Among Latino and AAPI individuals, the same overall pattern was observed with larger and less precise effect estimates than other groups. Among AIAN individuals, most results showed decreased odds of late-stage CRC in less advantaged areas, but the imprecise estimates included the null.

**Figure 2:**
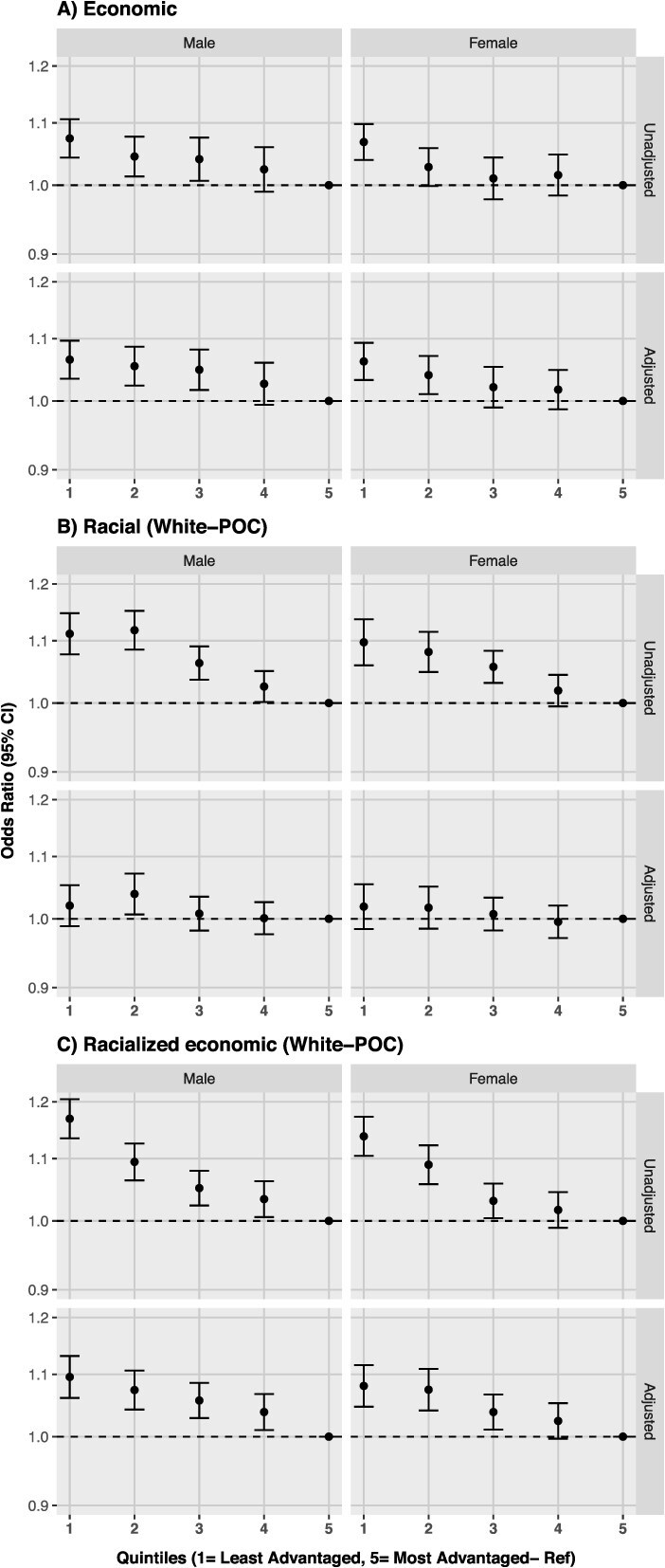
Odds of late-stage colorectal cancer by quintiles of residential segregation, stratified by sex in the United States, 2009-2017. Note: Residential segregation is based on the ICE. Panel A uses a measure of economic segregation, panel B uses a measure of racial segregation (White–people of color), and panel C uses a measure of racialized economic segregation (White–people of color). Quintile 1—least advantaged, quintile 5—Most advantaged (reference). Adjusted models included: age at diagnosis, race/ethnicity, census region, and year of diagnosis. Abbreviation: ICE, Index of Concentration at the Extremes.

**Figure 3:**
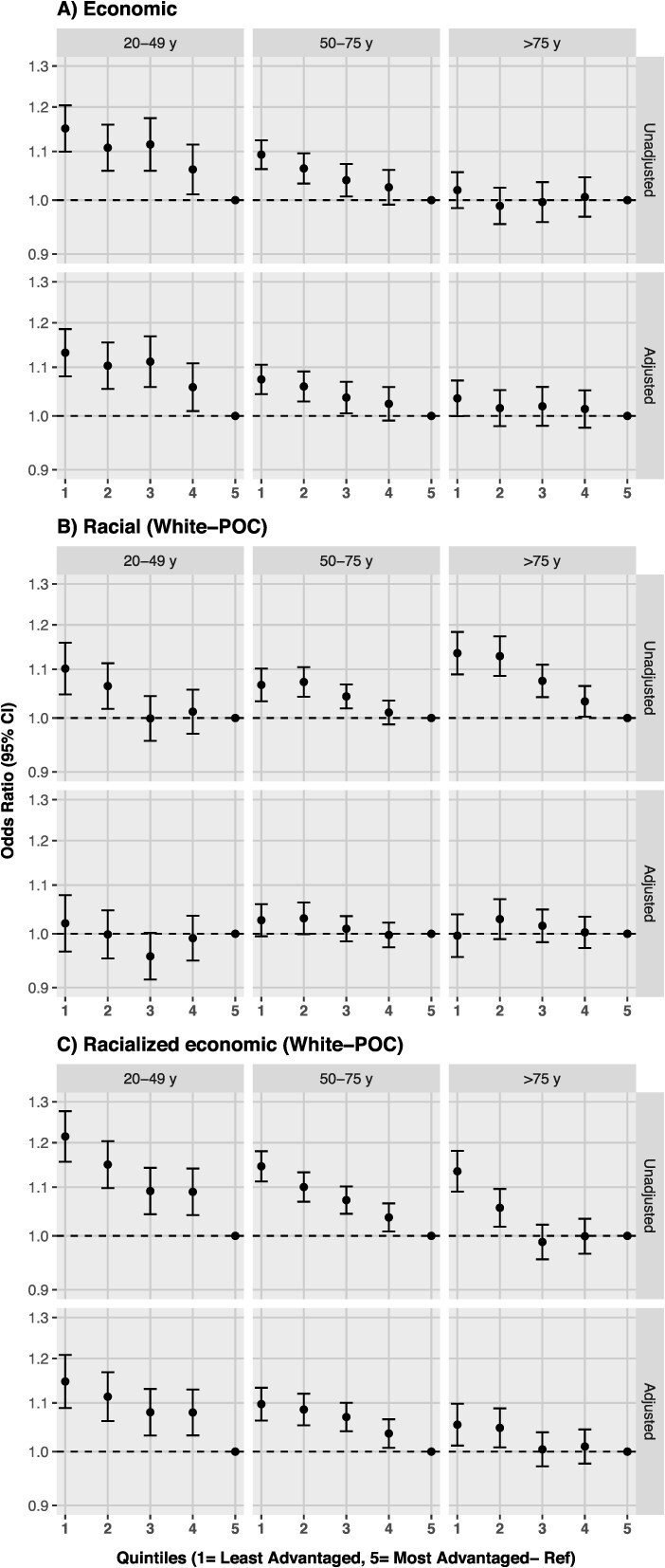
Odds of late-stage colorectal cancer by quintiles of residential segregation, stratified by age in the United States, 2009-2017. Note: Residential segregation is based on the ICE. Panel a uses a measure of economic segregation, panel B uses a measure of racial segregation (White–people of color), and panel C uses a measure of racialized economic segregation (White–people of color). Quintile 1—least advantaged, quintile 5—Most advantaged (reference). Adjusted models included: sex, race/ethnicity, census region, and year of diagnosis. Abbreviation: ICE, Index of Concentration at the Extremes.

In the economic segregation analyses stratified by sex, results mirrored the overall analysis for both females and males (Figure 2A). In age-stratified analyses, the gradient of increased odds of late-stage CRC in less advantaged counties compared to the most advantaged ones was clear for the 20-49 and 50-75 age groups, with stronger associations among the younger individuals (Figure 3A).

### Racial segregation

In the unadjusted racial segregation analysis overall, individuals residing in less advantaged counties at diagnosis (ie, more concentrated POC segregated counties) had increased odds of late-stage CRC compared to their counterparts living in the most advantaged counties (OR [95% CI]: Q1, 1.11 [1.08-1.14]; Q2, 1.11 [1.08-1.14]; Q3, 1.07 [1.05-1.09]; Q4, 1.02 [1.01-1.04]; Q5, reference; Figure 1B). In the adjusted analysis, estimates were attenuated, and some included the null, but the pattern remained.

In the racial segregation analysis stratified by individual race/ethnicity, findings were very similar in unadjusted and adjusted models with some attenuation. Among White and AAPI individuals, the majority of estimates show increased odds of late-stage CRC in less advantaged counties and included the null. Among Black individuals, increased odds of late-stage CRC were observed in less advantaged counties (OR [95% CI]: Q1, 1.06 [0.98-1.14]; Q2, 1.08 [1.01-1.16]; Q3, 1.04 [0.97-1.12]; Q4, 1.02 [0.95-1.10]; Q5, reference), and the opposite was found among Latino individuals (OR [95% CI]: Q1, 0.97 [0.87-1.07]; Q2, 0.99 [0.89-1.09]; Q3, 0.88 [0.80-0.98]; Q4, 0.93 [0.84-1.05]; Q5, reference). Among AIAN individuals, similar to economic segregation, we observed decreased odds of late-stage CRC in less advantaged counties, but estimates were unstable.

In the crude racial segregation analysis stratified by sex and by age, results were similar to the overall analysis (ie, increased odds of late-stage CRC in less advantaged counties) and estimates mostly attenuated in adjusted models (Figures 2B and 3B).

### Racialized economic segregation

For racialized economic segregation, variables included in the adjusted models attenuated estimates obtained in the crude analyses, but the general patterns persisted. Overall, in adjusted models, individuals living in less advantaged counties (ie, more concentrated low-income POC counties) had increased odds of late-stage CRC as compared to individuals residing in most advantaged counties (OR [95% CI]: Q1, 1.09 [1.06-1.12]; Q2, 1.08 [1.05-1.11]; Q3, 1.05 [1.02-1.07]; Q4, 1.03 [1.01-1.05]; Q5, reference; Figure 1C). In analyses stratified by race/ethnicity, estimates for White individuals aligned with the overall findings. Among racial/ethnic minoritized individuals, the same pattern with larger but less precise estimates were observed.

Results stratified by sex do not show differences among groups (Figure 2C), and by age, findings evidence the same overall pattern in all age groups with stronger associations in younger groups (Figure 3C).

### Sensitivity analyses

In Table S1, we compare individuals’ characteristics by CRC stage at diagnosis. We found that those with unknown stage were more likely to be older (mean age: unknown, 73 years; not late, 67 years; late, 65 years; % of cases ≥80 years: unknown, 39%; not late, 20%; late, 18%) and to reside in the South region of the United States (unknown, 42%; not late, 37%; late, 38%). We also observed that individuals with unknown stage had slightly higher percentages of residing in less advantaged counties. No major differences were observed in other characteristics.

In models including individuals with unknown stage at diagnosis, we observed that findings of the two different assumptions were consistent with the main analysis. Because individuals with unknown stage were more likely to reside in less advantaged counties, the least conservative scenario (all unknown cases treated as late) resulted in stronger associations across metrics of residential segregation (Figures S10-S12).

The effect estimates obtained from Poisson regression models were slightly smaller in magnitude than those observed in the main analyses, but patterns of associations for all ICE measures across all racial/ethnic groups remained (Figure S13).

## Discussion

In this population-based study of all CRC cases diagnosed in the United States during 2009-2017, we assessed the association between residential segregation and late-stage CRC. We observed that overall, people living in less advantaged areas, measured by quintiles of economic, racial/ethnic, and racialized economic segregation had increased odds of a late-stage diagnosis compared to people residing in more advantaged areas (ie, counties with higher concentration of high-income individuals, White individuals, or high-income White individuals). In general, stronger associations were identified for measures of residential segregation that included information about income, particularly racialized economic segregation. These results confirm findings of a previous study using 2009-2013 SEER data restricted to White and Black patients,^22^ suggesting that by incorporating these two dimensions of residential segregation and not only focusing on the racial aspect as most prior research, we were able to capture inequities in late-stage CRC that otherwise might have been missed.

When stratifying the analyses by demographic characteristics, we observed that results were not homogenous in all groups, with larger effect estimates and more clear gradients among younger age groups and diverging patterns by race/ethnicity. Stronger associations in individuals at younger ages, especially those with <50 years or early-onset cases, might be related to the fact that based on established guidelines during the study period CRC screening was not recommended (with the exception of high-risk individuals).^32^ Among the age group for which CRC screening was recommended (50-75 years),^32^ national estimates showed that younger individuals had lower uptake.^33^ Hence, it is possible that the majority of young people were diagnosed only when they presented symptoms that required a clinical intervention. Individuals residing in less advantaged areas may have had limited access to health care, potentially leading to delayed diagnoses. On the other hand, the indication of a colonoscopy might have allowed people residing in less advantaged areas but with ages in which screening was recommended, the opportunity to receive a diagnostic test, reducing the differences in odds of a late-stage disease within this group.^22^ Among the oldest age group (>75 years; screening not recommended but offered depending on circumstances of individuals^32^) odds of late-stage CRC were still significantly higher in people residing in less advantaged counties, highlighting the persistent contribution of residential segregation even in the presence of health insurance coverage (available through Medicare).

The distinct patterns observed by race/ethnicity reflect how the interplay of the social environment, the health care system, and individual characteristics may affect groups differently. The lack of an association for economic segregation among Black patients suggests that Black people residing in more advantaged areas do not benefit to the same extent as other racial/ethnic groups do from having better access to resources, perhaps due to additional barriers in the health care system (eg, discrimination, mistrust) that result in them being less likely to seek care and when they do less likely to receive high-quality care, leading to later diagnoses. For racial segregation, we found that Black people residing in less advantaged areas had higher odds of late-stage CRC; the opposite was observed among Latino people, and no clear patterns were seen in other groups. The apparent benefit of residential segregation documented among Latino patients could be attributed to greater social support and social cohesion often present in Latino communities, especially considering what it takes to undergo a colonoscopy (preparation, transportation, company), and the help needed before and after the procedure.^19^^,^^34^^,^^35^ Interestingly, for economic and racialized economic segregation, this “protective effect” was not observed. These findings could be explained by the inclusion of information about income in the segregation measures, suggesting that among Latino individuals, economic disadvantage may limit or even outweigh protective community factors.

Previous research evaluating the association between racial segregation and late-stage CRC have shown conflicting results. For example, using USCS data for 2004-2009 Mobley et al. found that more segregated communities, based on the isolation index, had lower likelihoods of late-stage CRC.^19^ Using the same dataset for more recent years (2008-2012) and employing the index of dissimilarity (IoD) measure of segregation, Moss et al. observed lower rates of late-stage CRC in highly segregated counties.^21^ In contrast, Poulson et al., analyzing 2005-2015 SEER-18 data and using IoD, found higher risk of late-stage CRC among White and Black patients residing in more segregated areas.^20^ Incongruities in these results could be explained by differences in how the studies were conducted (ie, differences in: data sources, measures of segregation, years of diagnoses included, and units of analysis).

In this study we followed the approach employed by Scally et al.^22^: individual-level cancer registry data was used to ascertain late-stage CRC, and economic, racial and realized economic segregation was measured at the county level using ICE. In their analysis, they used data from SEER-18 for years 2009-2013 (*n* = 187 843), evaluated racial and racialized economic segregation based on ACS estimates of White and Black individuals, and presented findings by age (<50 years, ≥50 years) and race (White, Black). In our analysis, we used USCS data for years 2009-2017 (*n* = 1 241 892), and created measures of racial and racialized economic segregation beyond White and Black populations (ie, POC, Latino, and AAPI populations). Because we had access to all CRC cases in the United States, we were able to test the associations of interest in additional subgroups (sex, male, female; age, 20-49 years; 50-75 years, >75 years; and race/ethnicity: White, Black, Latino, AAPI, AIAN). Our study confirmed their findings for economic and racialized economic segregation while showing clearer gradients (ie, monotonically increasing ORs in less advantaged areas). We also replicated their finding of age as an effect modifier. Additionally, we observed significant associations for racial segregation, and identified nuances in racial/ethnic groups that had not been previously described. A similarity between previous research and our study is the small size of effect estimates, a common observation in studies evaluating area-based exposures on health.^36^ Although the effect sizes of area-level indicators are often smaller in magnitude compared to individual-level characteristics, they provide useful information on aspects that may drive differences in health and are potentially modifiable. Moreover, even small effect sizes at the area level can have a large impact due to the high number of individuals who may be affected.

Overcoming the long-lasting effects of institutionalized racism in the United States, evident in the existing inequities and burden of a preventable disease like CRC, will require concerted efforts at multiple levels, but especially initiatives that consider the social environment of individuals.^7^^,^^37^^-^^39^ Considering that risk factors for CRC cluster geographically and are shaped by the social and built environments (eg, limited access to green spaces and recreational areas that support physical activity, food deserts, and food swamps that restrict access to affordable, healthy food), improving access to timely screening in these underserved areas should be prioritized. In a recent review, looking at how social determinants of health were considered in cancer screening interventions in the United States, authors found that most interventions focused on factors at the individual level (eg, health literacy, language barriers), and there was a lack of interventions addressing structural factors at the health care system or community levels, including the effects of residential segregation.^40^ Health policies at national and state levels that improve access to high-quality and continuous care could help in further reducing the burden of CRC in the United States. For example, evidence suggests that Medicaid expansion has increased both CRC screening and early stage CRC diagnoses and decreased late-stage CRC in the United States.^41^^,^^42^ Also, Delaware is an example of a state with programs that could be initiated elsewhere to reduce inequities in CRC. After creating partnerships in predominantly Black communities, the state implemented a combination of strategies and addressed barriers at different points of health care (health insurance coverage, patient navigation), increasing CRC screening uptake and reducing CRC late-stage diagnoses and mortality.^43^ At the community level, multisector partnerships between public health departments, academic institutions, and healthcare systems could improve access to preventive care services, including CRC screening and timely follow-up, in less advantaged areas.^44^^,^^45^ Also, coordination between community health centers and neighborhood-based social networks could help in disseminating information about CRC screening and providing resources that support individuals in these areas.^44^^,^^46^

There are several limitations to this study. First, individual-level data obtained from cancer registries did not enable us to control for all key confounding factors (eg, individual socioeconomic status, nativity status), presenting the possibility of residual confounding. Second, the lack of information on potential mediators such as health insurance coverage and participation in health care of patients precluded us from testing pathways in which residential segregation might impact early detection of CRC. Third, the cross-sectional study design, with measures of residential segregation at one point in time, might lead to misclassification of the exposure. Considering the latency period of CRC, individuals' county of residence at the time of diagnosis may differ from county of residence during key moments in the years prior to diagnosis. Fourth, the measures of residential segregation were calculated at the county level potentially masking existing inequities at smaller geographies in highly heterogeneous areas in the United States. Finally, the outcome variable was unknown for 97 687 cases. NPCR registries contributed 55% of all cases in this study (*n* = 684 981) but accounted for 58% of those with unknown stage (*n* = 56 774), reflecting lower completeness and specificity of information compared to SEER registries, as demonstrated in prior research.^47^ If reasons for unknown stage at diagnosis were also related to residential segregation, excluding those observations from the analysis could introduce selection bias. However, the main results were robust to several sensitivity analyses, including individuals with unknown stage in worst- and best-case scenarios.

In summary, the present study provides further evidence that place matters for prevention and early detection of CRC. Moreover, this study suggests the harmful consequences of institutionalized racism in the United States, with laws and policies that dictated where individuals could live, also playing a key role in contributing to the conditions for receiving timely preventive care. Continued efforts and resources are needed to support initiatives directed at eliminating the effects of residential segregation on CRC prevention and detection.

## Supplementary material

Supplementary material is available at the *American Journal of Epidemiology* online.

## Supplementary Material

Web_Material_kwaf285

## Data Availability

Although the United States Cancer Statistics database is publicly available, this study required using geographical identifiers, which can only be accessed through Federal Statistical Research Data Centers operated by the US Census Bureau.
