# Supplementary material for: Residential segregation and late-stage colorectal cancer in the United States: a population-based study of 1.2 million adults
Source: Am J Epidemiol. 2025 Dec 24;195(4):1163–74. doi: 10.1093/aje/kwaf285 (PMC13066339; doi:10.1093/aje/kwaf285)
Supplement: Web_Material_kwaf285 [file web_material_kwaf285.docx]

**Supplementary Material**

**Residential segregation and late-stage colorectal cancer in the United States: a population-based study of 1.2 million adults**

Eduardo J. Santiago-Rodríguez, Justin S. White, Zinzi D. Bailey, Isabel E. Allen, Robert A. Hiatt, Salma Shariff-Marco

**Table of Contents**

**Figure S1.** Distribution of economic segregation by the Index of Concentration at the Extremes (ICE), overall and stratified by race/ethnicity in the United States, 2009-2017

**Figure S2.** Distribution of racial segregation by the Index of Concentration at the Extremes (ICE), overall and stratified by race/ethnicity in the United States, 2009-2017

**Figure S3.** Distribution of racialized economic segregation by the Index of Concentration at the Extremes (ICE), overall and stratified by race/ethnicity in the United States, 2009-2017

**Figure S4.** Odds of late-stage colorectal cancer by quintiles of racial segregation, overall and stratified by race/ethnicity in the United States, 2009-2017

**Figure S5.** Odds of late-stage colorectal cancer by quintiles of racialized economic segregation, overall and stratified by race/ethnicity in the United States, 2009-2017

**Figure S6.** Odds of late-stage colorectal cancer by quintiles of racial segregation, stratified by sex in the United States, 2009-2017

**Figure S7.** Odds of late-stage colorectal cancer by quintiles of racialized economic segregation, stratified by sex in the United States, 2009-2017

**Figure S8.** Odds of late-stage colorectal cancer by quintiles of racial segregation, stratified by age in the United States, 2009-2017

**Figure S9.** Odds of late-stage colorectal cancer by quintiles of racialized economic segregation, stratified by age in the United States, 2009-2017

**Figure S10.** Odds of late-stage colorectal cancer by quintiles of residential segregation in main analysis (overall), and two scenarios of sensitivity analysis

**Figure S11.** Odds of late-stage colorectal cancer by quintiles of racial segregation in main analysis (overall), and two scenarios of sensitivity analysis

**Figure S12.** Odds of late-stage colorectal cancer by quintiles of racialized economic segregation in main analysis (overall), and two scenarios of sensitivity analysis

**Figure S13.** Effect estimates of late-stage colorectal cancer by quintiles of residential segregation using two different specifications of multilevel modeling

**Table S1.** Characteristics of individuals with colorectal cancer by stage at diagnosis in the United States 2009-2017

**Figure S1.** Distribution of economic segregation by the Index of Concentration at the Extremes (ICE), overall and stratified by race/ethnicity in the United States, 2009-2017


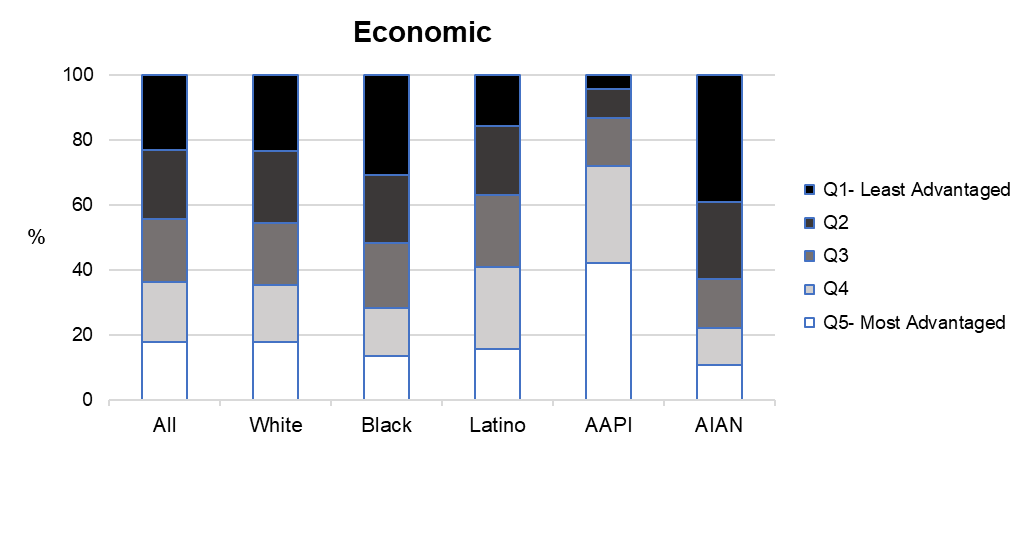


**Figure S2.** Distribution of racial segregation by the Index of Concentration at the Extremes (ICE), overall and stratified by race/ethnicity in the United States, 2009-2017


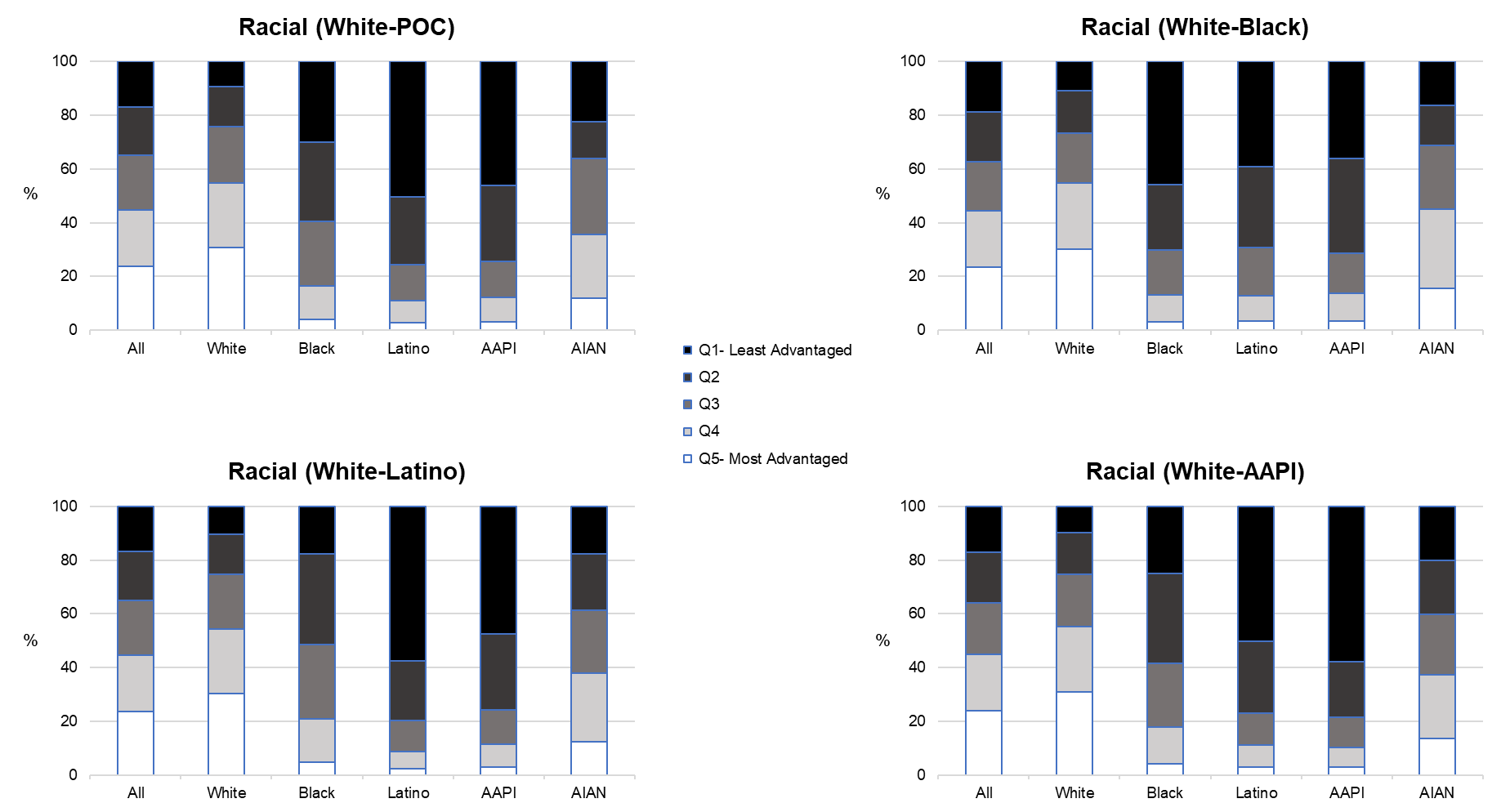


**Figure S3.** Distribution of racialized economic segregation by the Index of Concentration at the Extremes (ICE), overall and stratified by race/ethnicity in the United States, 2009-2017


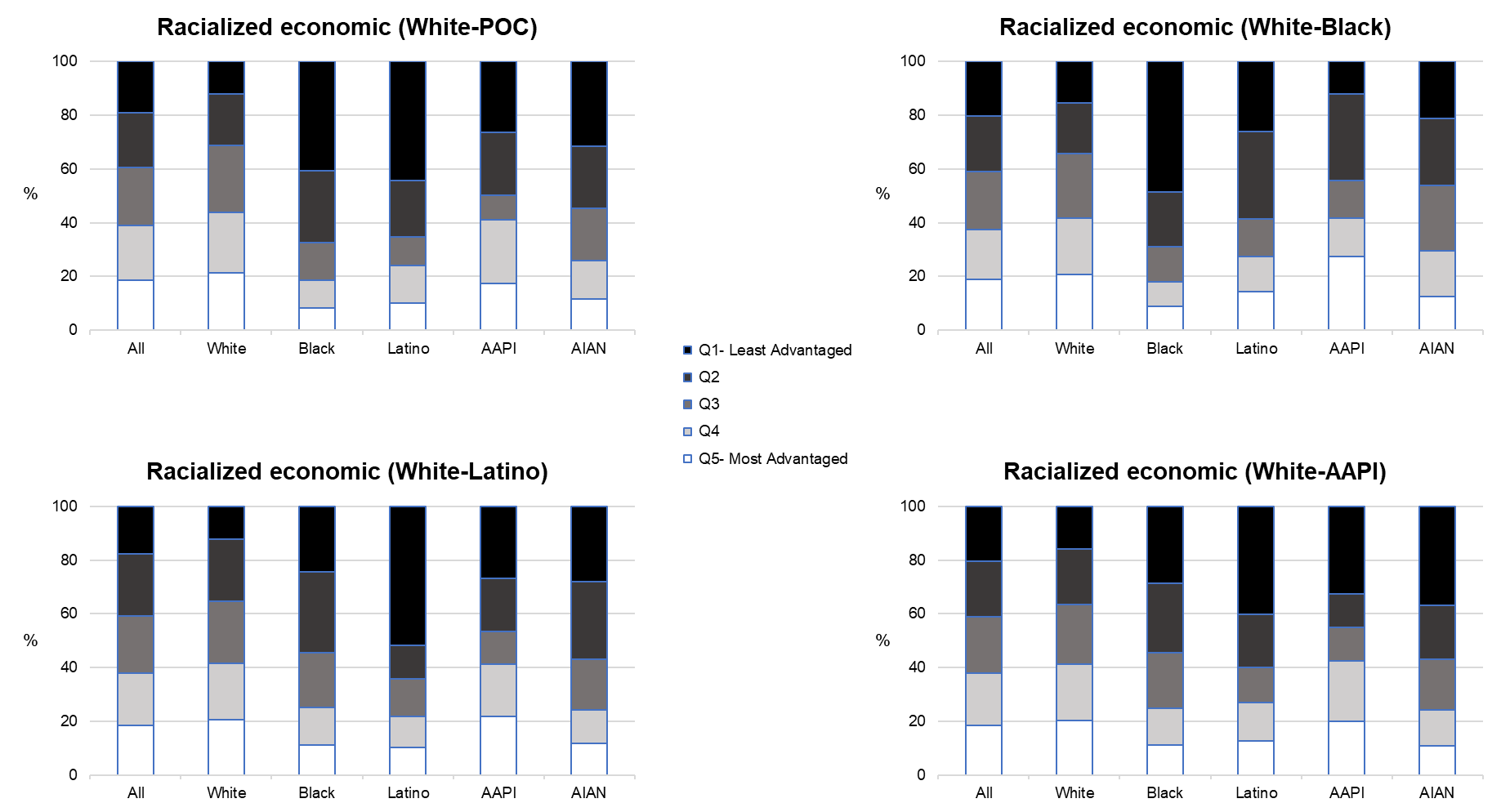


**Figure S4.** Odds of late-stage colorectal cancer by quintiles of racial segregation, overall and stratified by race/ethnicity in the United States, 2009-2017


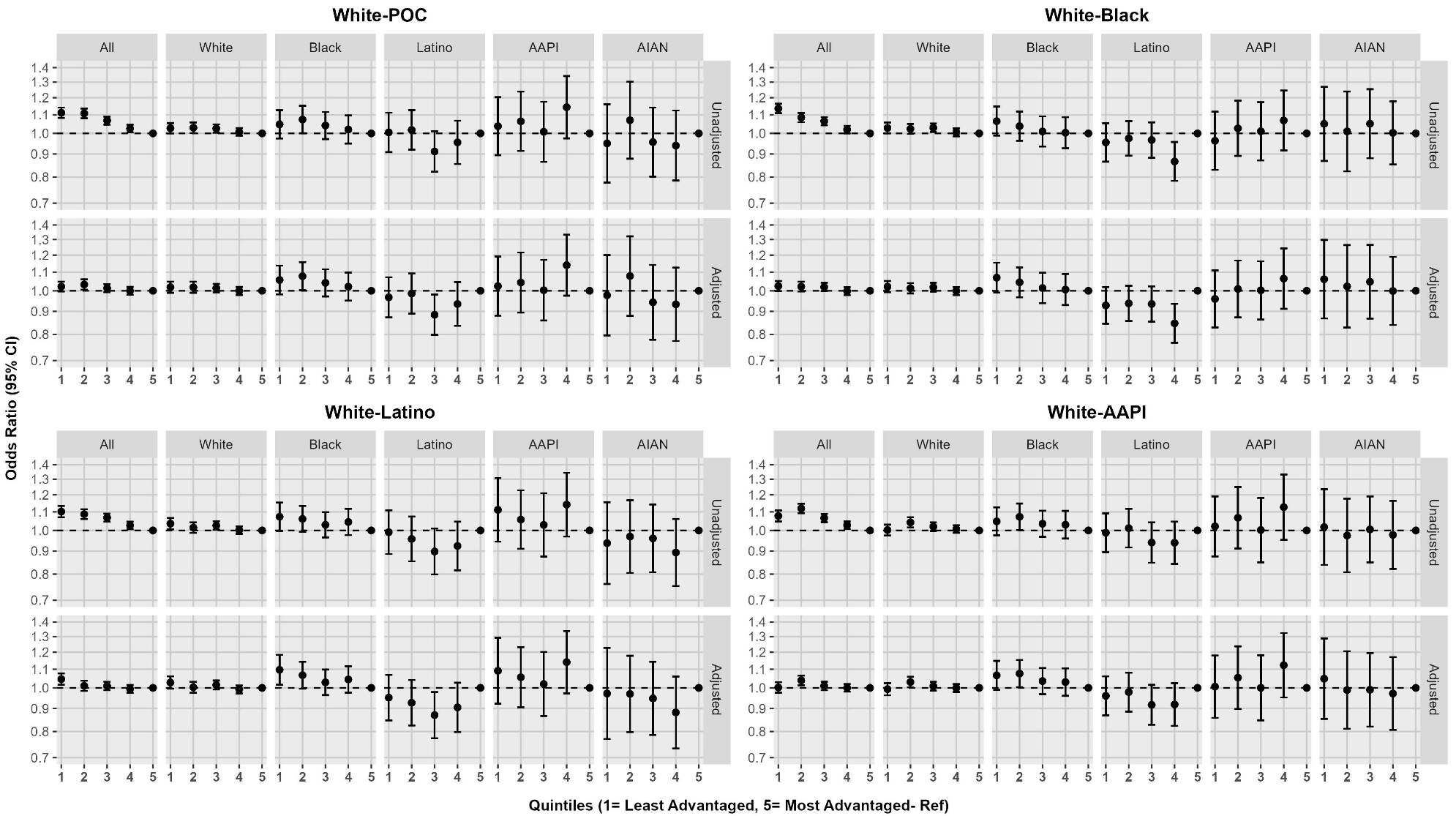


Note: Racial segregation is based on the Index of Concentration at the Extremes (ICE). Quintile 1- Least advantaged, Quintile 5- Most advantaged (Reference). Adjusted models included: age at diagnosis, sex, race/ethnicity, census region and year of diagnosis.

**Figure S5.** Odds of late-stage colorectal cancer by quintiles of racialized economic segregation, overall and stratified by race/ethnicity in the United States, 2009-2017


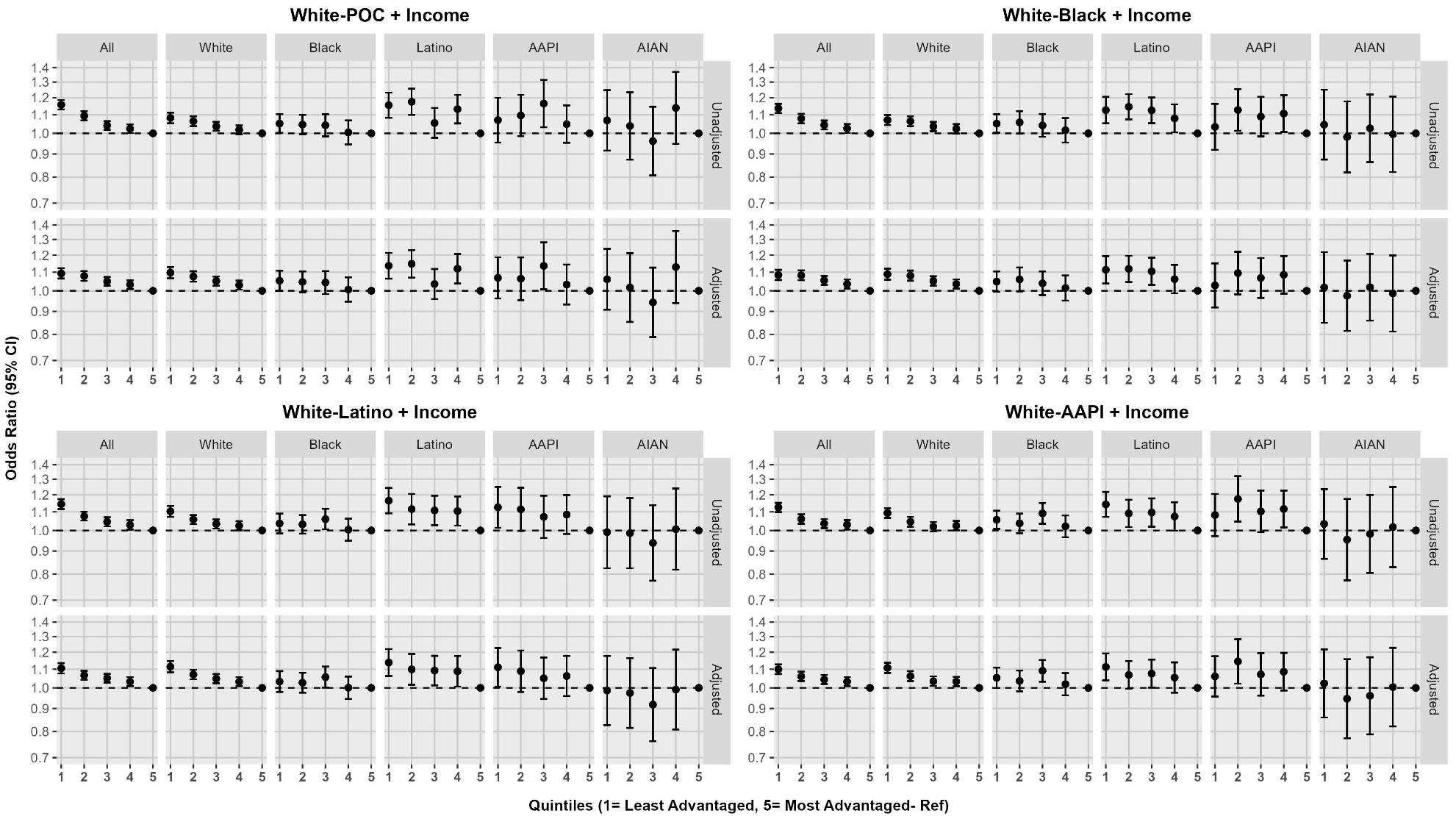


Note: Racialized economic segregation is based on the Index of Concentration at the Extremes (ICE). Quintile 1- Least advantaged, Quintile 5- Most advantaged (Reference). Adjusted models included: age at diagnosis, sex, race/ethnicity, census region and year of diagnosis.

**Figure S6.** Odds of late-stage colorectal cancer by quintiles of racial segregation, stratified by sex in the United States, 2009-2017


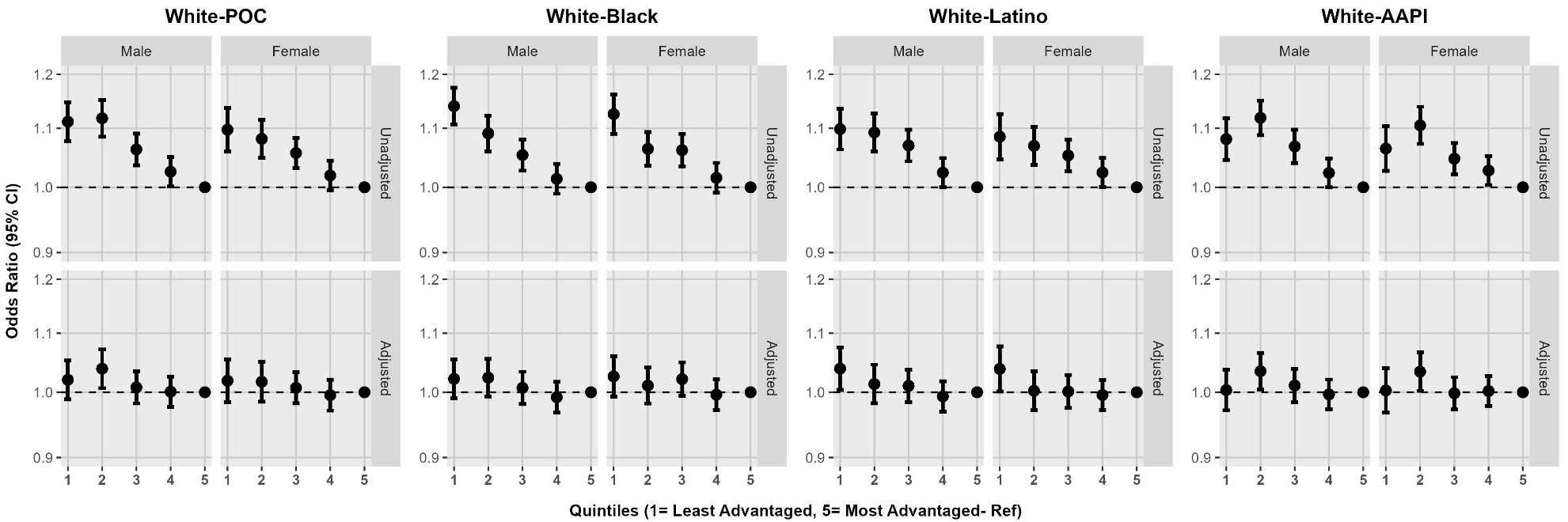


Note: Racial segregation is based on the Index of Concentration at the Extremes (ICE). Quintile 1- Least advantaged, Quintile 5- Most advantaged (Reference). Adjusted models included: age at diagnosis, race/ethnicity, census region and year of diagnosis.

**Figure S7.** Odds of late-stage colorectal cancer by quintiles of racialized economic segregation, stratified by sex in the United States, 2009-2017


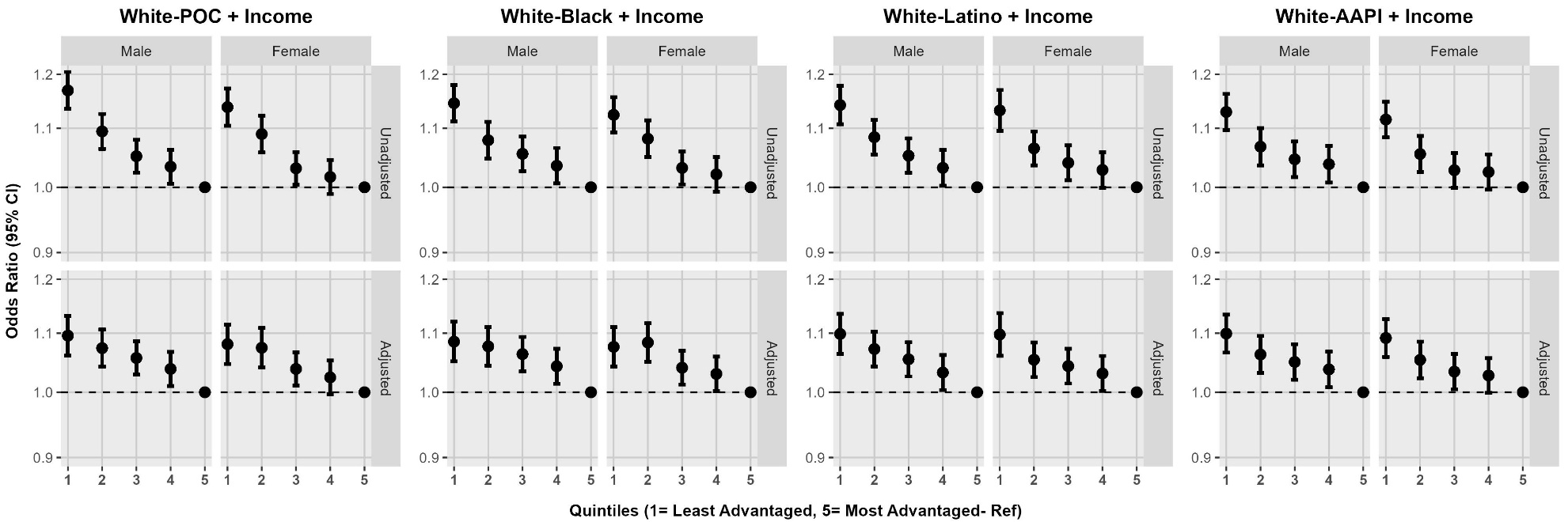


Note: Racialized economic segregation is based on the Index of Concentration at the Extremes (ICE). Quintile 1- Least advantaged, Quintile 5- Most advantaged (Reference). Adjusted models included: age at diagnosis, race/ethnicity, census region and year of diagnosis.

**Figure S8.** Odds of late-stage colorectal cancer by quintiles of racial segregation, stratified by age in the United States, 2009-2017


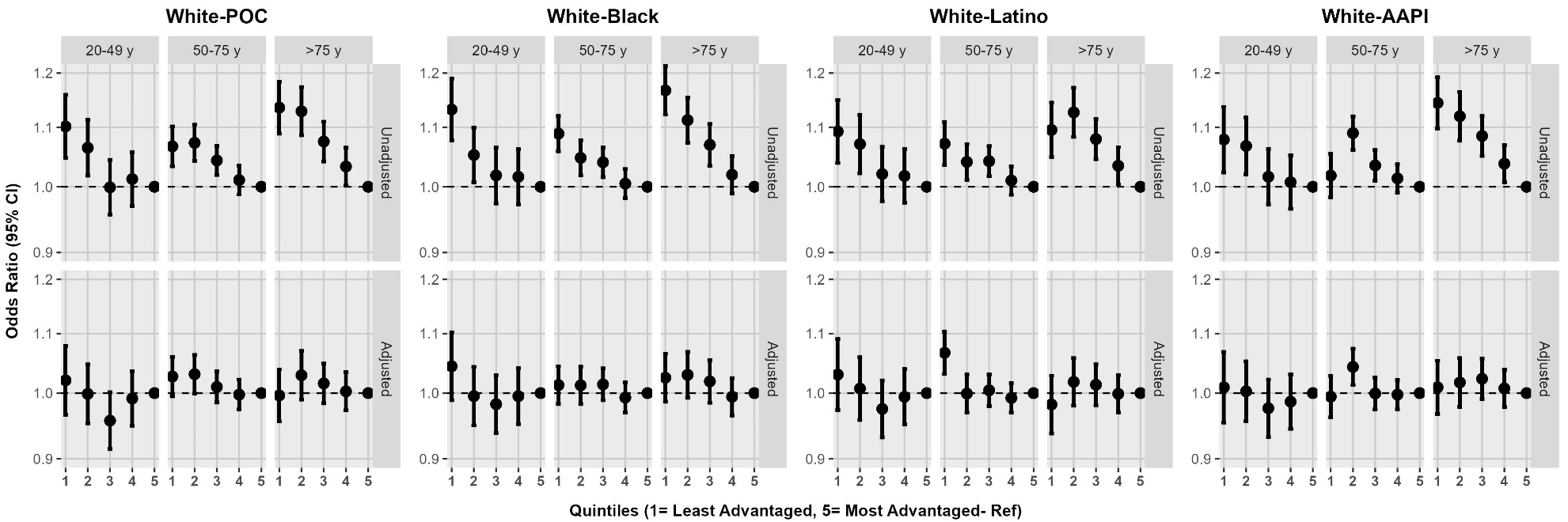


Note: Racial segregation is based on the Index of Concentration at the Extremes (ICE). Quintile 1- Least advantaged, Quintile 5- Most advantaged (Reference). Adjusted models included: sex, race/ethnicity, census region and year of diagnosis.

**Figure S9.** Odds of late-stage colorectal cancer by quintiles of racialized economic segregation, stratified by age in the United States, 2009-2017


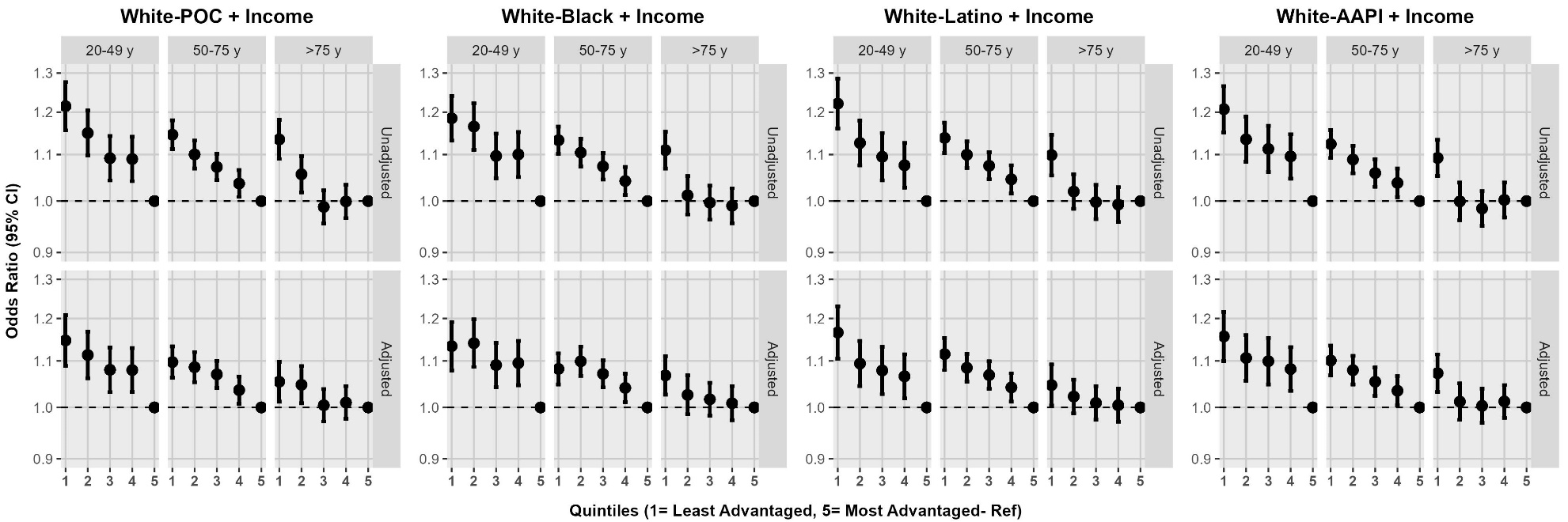


Note: Racialized economic segregation is based on the Index of Concentration at the Extremes (ICE). Quintile 1- Least advantaged, Quintile 5- Most advantaged (Reference). Adjusted models included: sex, race/ethnicity, census region and year of diagnosis.

**Figure S10.** Odds of late-stage colorectal cancer by quintiles of residential segregation in main analysis (overall), and two scenarios of sensitivity analysis


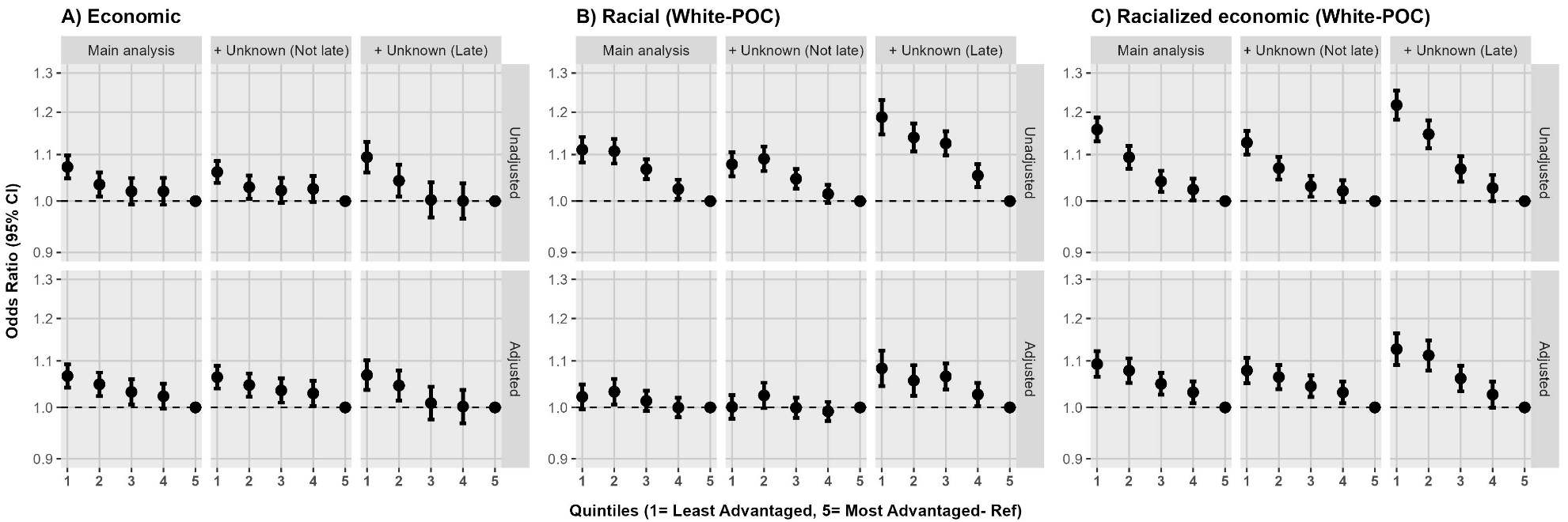


Note: Residential segregation is based on the Index of Concentration at the Extremes (ICE). Panel A uses a measure of economic segregation, Panel B uses a measure of racial segregation (White-People of Color), and Panel C uses a measure of racialized economic segregation (White-People of Color). Quintile 1- Least advantaged, Quintile 5- Most advantaged (Reference). Adjusted models included: age at diagnosis, sex, race/ethnicity, census region and year of diagnosis.

**Figure S11.** Odds of late-stage colorectal cancer by quintiles of racial segregation in main analysis (overall), and two scenarios of sensitivity analysis


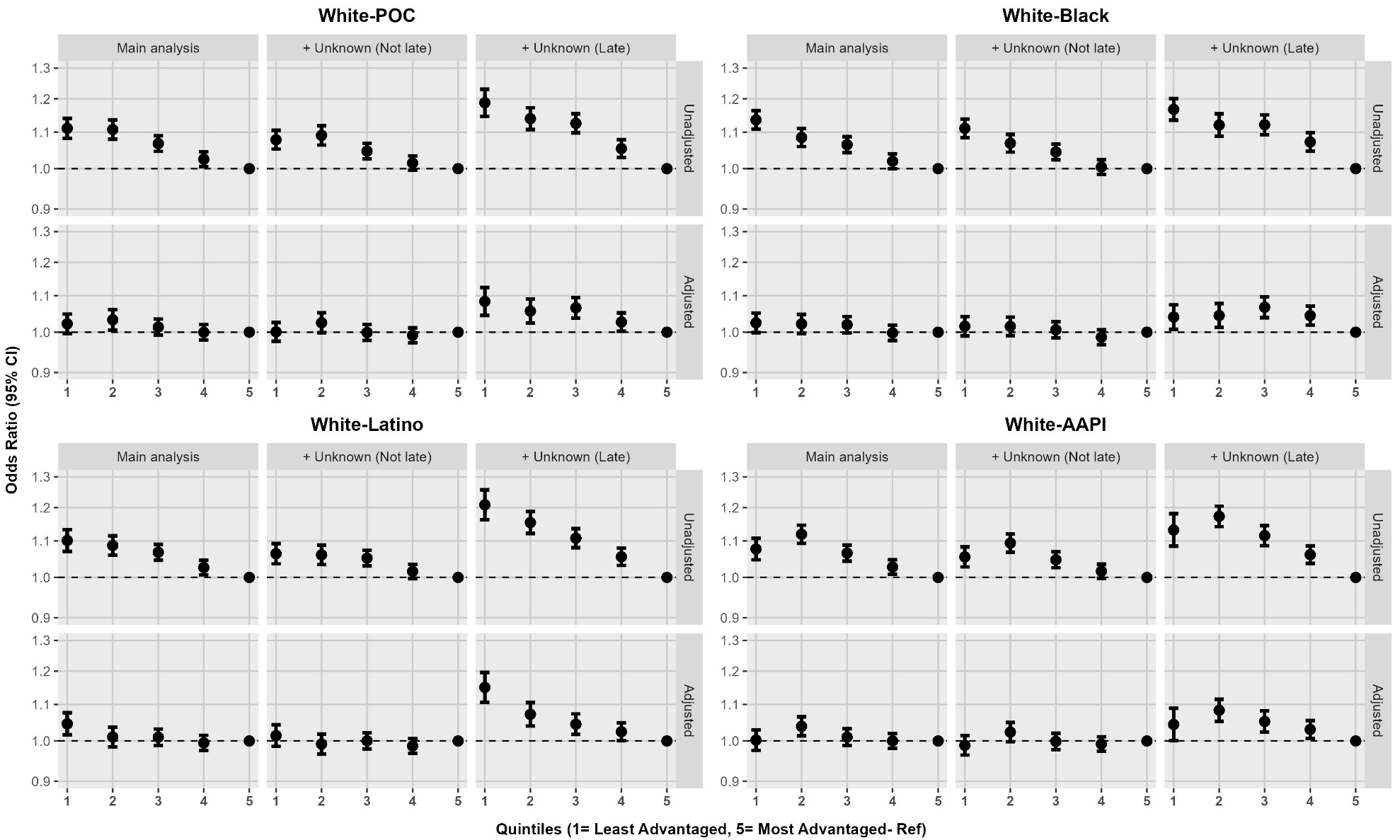


Note: Racial segregation is based on the Index of Concentration at the Extremes (ICE). Quintile 1- Least advantaged, Quintile 5- Most advantaged (Reference). Adjusted models included: age at diagnosis, sex, race/ethnicity, census region and year of diagnosis.

**Figure S12.** Odds of late-stage colorectal cancer by quintiles of racialized economic segregation in main analysis (overall), and two scenarios of sensitivity analysis


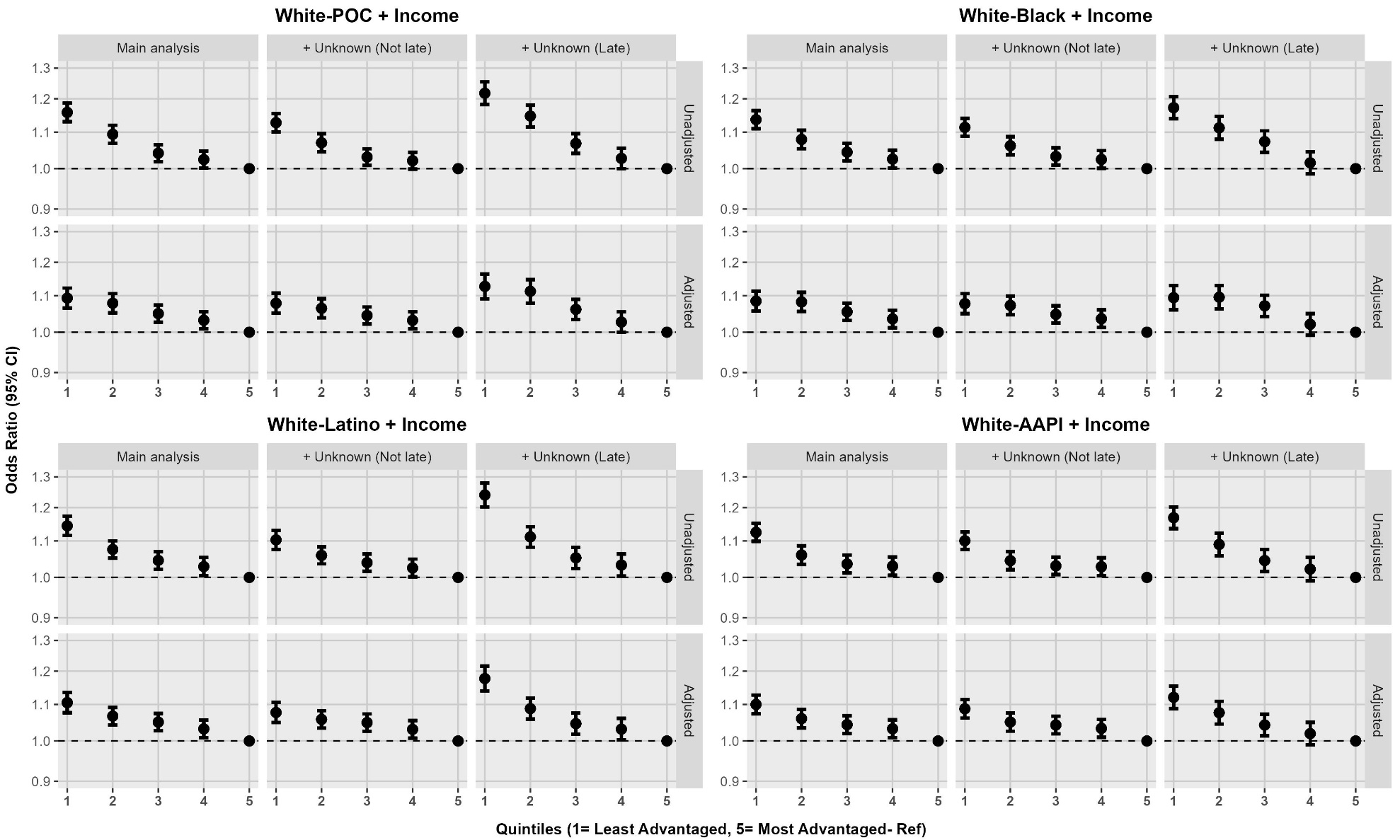
Note: Racialized economic segregation is based on the Index of Concentration at the Extremes (ICE). Quintile 1- Least advantaged, Quintile 5- Most advantaged (Reference). Adjusted models included: age at diagnosis, sex, race/ethnicity, census region and year of diagnosis.

**Figure S13.** Effect estimates of late-stage colorectal cancer by quintiles of residential segregation using two different specifications of multilevel modeling


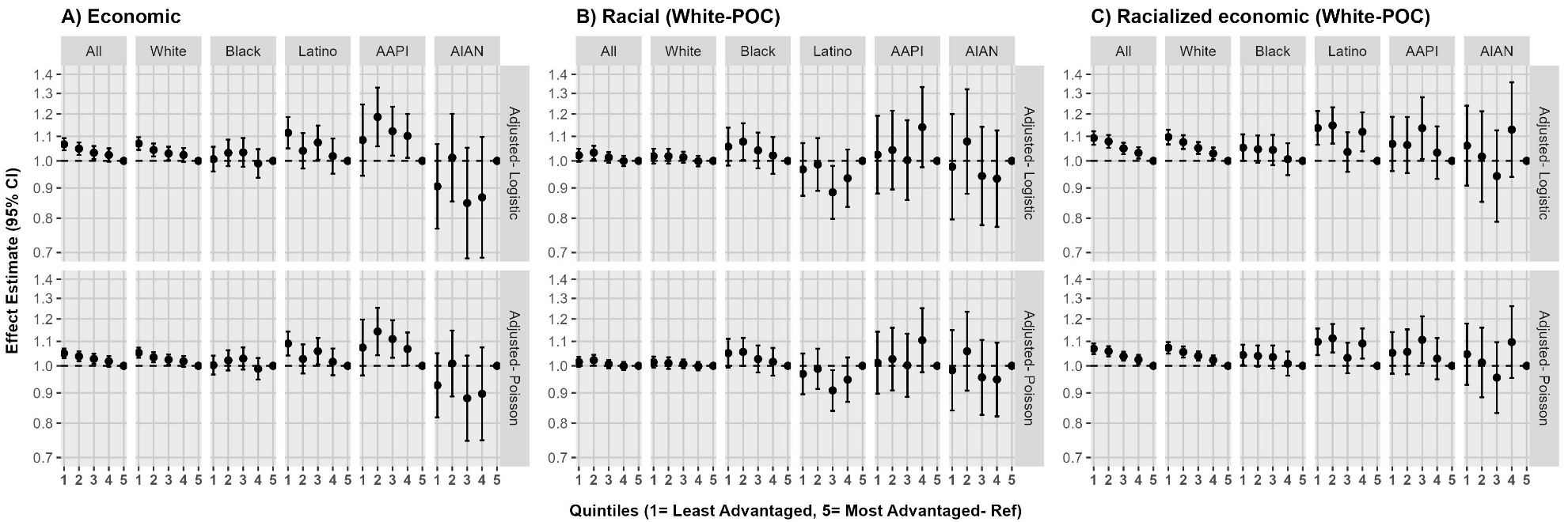


Note: Logistic regression, main analysis (top row); Poisson regression, sensitivity analysis (bottom row). Residential segregation is based on the Index of Concentration at the Extremes (ICE). Panel A uses a measure of economic segregation, Panel B uses a measure of racial segregation (White-People of Color), and Panel C uses a measure of racialized economic segregation (White-People of Color). Quintile 1- Least advantaged, Quintile 5- Most advantaged (Reference). Models included: age at diagnosis, sex, race/ethnicity, census region and year of diagnosis.

**Table S1.** Characteristics of individuals with colorectal cancer by stage at diagnosis in the United States 2009-2017

| Characteristic | All,  n = 1,339,579 | Not Late,  n = 978,110 | Late,  n = 263,782 | Unknown,  n = 97,687 |
| --- | --- | --- | --- | --- |
| Age at diagnosis; mean (SD) | 67.0 (14.1) | 66.9 (13.8) | 65.4 (14.1) | 72.6 (15.3) |
| Age at diagnosis, y; n (%) |  |  |  |  |
| 20-24 | 2,934 (0.2) | 2,338 (0.2) | 427 (0.2) | 169 (0.2) |
| 25-29 | 5,754 (0.4) | 4,218 (0.4) | 1,231 (0.5) | 305 (0.3) |
| 30-34 | 10,861 (0.8) | 7,612 (0.8) | 2,686 (1) | 563 (0.6) |
| 35-39 | 19,372 (1.5) | 13,364 (1.4) | 5,049 (1.9) | 959 (1) |
| 40-44 | 36,110 (2.7) | 25,270 (2.6) | 9,135 (3.5) | 1,705 (1.8) |
| 45-49 | 66,107 (4.9) | 45,902 (4.7) | 17,080 (6.5) | 3,125 (3.2) |
| 50-54 | 126,325 (9.4) | 94,789 (9.7) | 24,680 (9.4) | 6,856 (7) |
| 55-59 | 137,738 (10.3) | 99,795 (10.2) | 30,464 (11.6) | 7,479 (7.7) |
| 60-64 | 158,943 (11.9) | 115,605 (11.8) | 34,486 (13.1) | 8,852 (9.1) |
| 65-69 | 175,712 (13.1) | 131,813 (13.5) | 34,020 (12.9) | 9,879 (10.1) |
| 70-74 | 161,872 (12.1) | 122,522 (12.5) | 29,836 (11.3) | 9,514 (9.7) |
| 75-79 | 151,883 (11.3) | 115,288 (11.8) | 26,558 (10.1) | 10,037 (10.3) |
| 80-84 | 135,023 (10.1) | 100,109 (10.2) | 23,077 (8.8) | 11,837 (12.1) |
| ≥85 | 150,945 (11.3) | 99,485 (10.2) | 25,053 (9.5) | 26,407 (27) |
| Sex; n (%) |  |  |  |  |
| Male | 702,209 (52.4) | 512,700 (52.4) | 140,123 (53.1) | 49,386 (50.6) |
| Female | 637,370 (47.6) | 465,410 (47.6) | 123,659 (46.9) | 48,301 (49.4) |
| Race/ethnicity; n (%) |  |  |  |  |
| White | 997,372 (74.5) | 736,324 (75.3) | 192,058 (72.8) | 68,990 (70.6) |
| Black | 163,373 (12.2) | 113,164 (11.6) | 37,493 (14.2) | 12,716 (13) |
| Latino | 8,886 (0.7) | 6,199 (0.6) | 1,893 (0.7) | 794 (0.8) |
| Asian American/Pacific Islander | 48,956 (3.7) | 36,439 (3.7) | 9,064 (3.4) | 3,453 (3.5) |
| American Indian/Alaska Native | 110,967 (8.3) | 79,435 (8.1) | 22,581 (8.6) | 8,951 (9.2) |
| Unknown | 10,025 (0.8) | 6,549 (0.7) | 693 (0.3) | 2,783 (2.9) |
| Census region; n (%) |  |  |  |  |
| Midwest | 306,790 (22.9) | 227,005 (23.2) | 59,522 (22.6) | 20,263 (20.7) |
| Northeast | 261,722 (19.5) | 193,616 (19.8) | 50,923 (19.3) | 17,183 (17.6) |
| South | 504,109 (37.6) | 361,746 (37) | 101,146 (38.3) | 41,217 (42.2) |
| West | 266,958 (19.9) | 195,743 (20) | 52,191 (19.8) | 19,024 (19.5) |
| Year of diagnosis; n (%) |  |  |  |  |
| 2009 | 151,405 (11.3) | 112,749 (11.5) | 27,705 (10.5) | 10,951 (11.2) |
| 2010 | 147,316 (11) | 108,734 (11.1) | 28,032 (10.6) | 10,550 (10.8) |
| 2011 | 146,815 (11) | 107,914 (11) | 28,446 (10.8) | 10,455 (10.7) |
| 2012 | 145,797 (10.9) | 106,800 (10.9) | 28,622 (10.9) | 10,375 (10.6) |
| 2013 | 146,993 (11) | 106,841 (10.9) | 29,893 (11.3) | 10,259 (10.5) |
| 2014 | 149,287 (11.1) | 108,179 (11.1) | 30,288 (11.5) | 10,820 (11.1) |
| 2015 | 150,019 (11.2) | 108,711 (11.1) | 30,520 (11.6) | 10,788 (11) |
| 2016 | 151,448 (11.3) | 109,357 (11.2) | 30,223 (11.5) | 11,868 (12.2) |
| 2017 | 150,499 (11.2) | 108,825 (11.1) | 30,053 (11.4) | 11,621 (11.9) |
| ICE-Income; n (%) |  |  |  |  |
| Q1 | 301,255 (23.1) | 217,858 (22.9) | 60,929 (23.7) | 22,468 (23.5) |
| Q2 | 279,113 (21.4) | 202,544 (21.3) | 54,913 (21.4) | 21,656 (22.7) |
| Q3 | 254,196 (19.5) | 184,169 (19.4) | 50,303 (19.6) | 19,724 (20.7) |
| Q4 | 236,700 (18.1) | 174,628 (18.3) | 46,282 (18) | 15,790 (16.5) |
| Q5 | 233,282 (17.9) | 172,727 (18.2) | 44,720 (17.4) | 15,835 (16.6) |
| ICE- Race, White-People of Color; n (%) |  |  |  |  |
| Q1 | 223,672 (17.2) | 159,945 (16.8) | 45,005 (17.5) | 18,722 (19.6) |
| Q2 | 234,313 (18) | 170,032 (17.9) | 47,306 (18.4) | 16,975 (17.8) |
| Q3 | 267,690 (20.5) | 194,502 (20.4) | 52,536 (20.4) | 20,652 (21.6) |
| Q4 | 270,224 (20.7) | 198,534 (20.9) | 52,634 (20.5) | 19,056 (20) |
| Q5 | 308,647 (23.7) | 228,913 (24.1) | 59,666 (23.2) | 20,068 (21) |
| ICE- Race, White-Black; n (%) |  |  |  |  |
| Q1 | 245,300 (18.8) | 175,386 (18.4) | 50,090 (19.5) | 19,824 (20.8) |
| Q2 | 243,402 (18.7) | 176,553 (18.6) | 48,652 (18.9) | 18,197 (19.1) |
| Q3 | 240,300 (18.4) | 174,862 (18.4) | 47,280 (18.4) | 18,158 (19) |
| Q4 | 274,287 (21) | 201,566 (21.2) | 52,778 (20.5) | 19,943 (20.9) |
| Q5 | 301,257 (23.1) | 223,559 (23.5) | 58,347 (22.7) | 19,351 (20.3) |
| ICE- Race, White-Latino; n (%) |  |  |  |  |
| Q1 | 221,119 (17) | 158,987 (16.7) | 44,077 (17.1) | 18,055 (18.9) |
| Q2 | 242,031 (18.6) | 174,190 (18.3) | 48,150 (18.7) | 19,691 (20.6) |
| Q3 | 263,590 (20.2) | 192,159 (20.2) | 52,449 (20.4) | 18,982 (19.9) |
| Q4 | 274,360 (21) | 201,513 (21.2) | 53,618 (20.9) | 19,229 (20.1) |
| Q5 | 303,446 (23.3) | 225,077 (23.6) | 58,853 (22.9) | 19,516 (20.4) |
| ICE- Race, White- Asian American/Pacific Islander; n (%) | |  |  |  |
| Q1 | 222,923 (17.1) | 161,055 (16.9) | 44,378 (17.3) | 17,490 (18.3) |
| Q2 | 248,757 (19.1) | 178,502 (18.8) | 50,363 (19.6) | 19,892 (20.8) |
| Q3 | 249,656 (19.1) | 182,170 (19.1) | 49,106 (19.1) | 18,380 (19.3) |
| Q4 | 273,408 (21) | 200,568 (21.1) | 53,398 (20.8) | 19,442 (20.4) |
| Q5 | 309,802 (23.8) | 229,631 (24.1) | 59,902 (23.3) | 20,269 (21.2) |
| ICE- Race, White-People of Color + income; n (%) | |  |  |  |
| Q1 | 249,242 (19.1) | 177,969 (18.7) | 51,188 (19.9) | 20,085 (21) |
| Q2 | 270,013 (20.7) | 194,114 (20.4) | 54,022 (21) | 21,877 (22.9) |
| Q3 | 283,483 (21.7) | 206,978 (21.7) | 55,317 (21.5) | 21,188 (22.2) |
| Q4 | 262,341 (20.1) | 194,390 (20.4) | 51,075 (19.9) | 16,876 (17.7) |
| Q5 | 239,467 (18.4) | 178,475 (18.8) | 45,545 (17.7) | 15,447 (16.2) |
| ICE- Race, White-Black + income; n (%) |  |  |  |  |
| Q1 | 266,995 (20.5) | 190,845 (20.1) | 54,895 (21.4) | 21,255 (22.3) |
| Q2 | 272,722 (20.9) | 196,834 (20.7) | 54,348 (21.2) | 21,540 (22.6) |
| Q3 | 278,951 (21.4) | 203,965 (21.4) | 54,543 (21.2) | 20,443 (21.4) |
| Q4 | 241,480 (18.5) | 178,127 (18.7) | 46,997 (18.3) | 16,356 (17.1) |
| Q5 | 243,739 (18.7) | 181,671 (19.1) | 46,232 (18) | 15,836 (16.6) |
| ICE- Race, White-Latino + income; n (%) |  |  |  |  |
| Q1 | 236,386 (18.1) | 167,909 (17.6) | 47,777 (18.6) | 20,700 (21.7) |
| Q2 | 300,219 (23) | 217,115 (22.8) | 60,194 (23.4) | 22,910 (24) |
| Q3 | 278,647 (21.4) | 203,955 (21.4) | 54,616 (21.2) | 20,076 (21) |
| Q4 | 250,393 (19.2) | 184,912 (19.4) | 48,993 (19.1) | 16,488 (17.3) |
| Q5 | 238,901 (18.3) | 178,035 (18.7) | 45,567 (17.7) | 15,299 (16) |
| ICE- Race, White- Asian American/Pacific Islander + income; n (%) | |  |  |  |
| Q1 | 267,159 (20.5) | 191,521 (20.1) | 54,122 (21.1) | 21,516 (22.5) |
| Q2 | 275,659 (21.1) | 198,260 (20.8) | 55,119 (21.4) | 22,280 (23.3) |
| Q3 | 270,924 (20.8) | 198,454 (20.9) | 52,999 (20.6) | 19,471 (20.4) |
| Q4 | 251,788 (19.3) | 185,931 (19.5) | 49,321 (19.2) | 16,536 (17.3) |
| Q5 | 239,016 (18.3) | 177,760 (18.7) | 45,586 (17.7) | 15,670 (16.4) |
